# Recruitment of patients with de novo Parkinson disease: successful strategies in a randomized exercise clinical trial

**DOI:** 10.1186/s13063-018-2958-z

**Published:** 2018-11-14

**Authors:** Deborah A. Hall, Charity Moore, Cynthia Comella, the SPARX Study Group, B. Berman, C. L. Christiansen, B. Kluger, W. M. Kohrt, Ed Melanson, M. Schenkman, C. G. Moore, D. M. Corcos, C. Poon, A. Delitto, D. A. Josbeno, S. Jain

**Affiliations:** 1 Department of Neurological Sciences, Rush University, 1725 West Harrison Street, Suite 755, Chicago, IL 60612 USA; 2 Department of Physical Therapy, University of Pittsburgh, 100 Technology Drive, Suite 210, Pittsburgh, PA 15219 USA

**Keywords:** Parkinson disease, Clinical trial, Treadmill training, Recruitment, De novo

## Abstract

**Introduction:**

Recruitment of sufficient patients with Parkinson disease into clinical trials is a barrier to successful, timely study completion. Non-pharmacologic studies have shown to be even more challenging for recruitment, despite some studies focusing on de novo Parkinson disease populations. This paper describes successful recruitment techniques from a randomized exercise clinical trial in Parkinson disease.

**Methods:**

Several recruitment strategies were used to enroll de novo patients with Parkinson disease into a year-long clinical trial. Strategies focused on infrastructure included fast-track clinic scheduling, weekly research meetings, an established clinical repository, real-time clinic recruitment, and outreach to the community. The nature of the study facilitated recruitment by offering a wait-listed control group, exercise at a local fitness center with a paid membership, and collection of data by shipping equipment foregoing some visits. An experienced nurse study coordinator involved in recruitment and training of the principal investigator in recruitment of minorities enhanced overall recruitment. Finally, the patient population chosen for this study, patients with de novo Parkinson disease, may be more likely to enroll in an exercise study than patients with later stage disease.

**Results:**

Seventy-six patients with de novo Parkinson disease were successfully enrolled into the exercise clinical trial from a single site.

**Conclusion:**

Targeted recruitment strategies were successful in this study. Additional modifications to the study protocol, such as eliminating treadmill stress tests before randomization, travel to an urban downtown location for study visits, and a relatively healthy Parkinson disease population, may also have impacted this study. These strategies could all be adopted for other studies in Parkinson disease, neurodegenerative diseases, or other chronic disorders.

**Trial registration:**

Clinicaltrials.gov, NCT01506479. Registered on 10 January 2012.

**Electronic supplementary material:**

The online version of this article (10.1186/s13063-018-2958-z) contains supplementary material, which is available to authorized users.

## Background

Recruitment of adequate numbers of suitable patients for randomized clinical trials (RCTs) is frequently a major challenge in successful, timely completion of these studies [1]. Only 31% of RCTs complete recruitment on time and only a minority of RCTs recruit to the original target, even with an extension [2]. These problems are also seen in the recruitment of patients with Parkinson disease (PD) [1]. Recruitment for non-pharmacological intervention studies may be even more challenging, especially when the intervention is readily available to the patient, such as in physical therapy studies [3].

**Fig. 1:**
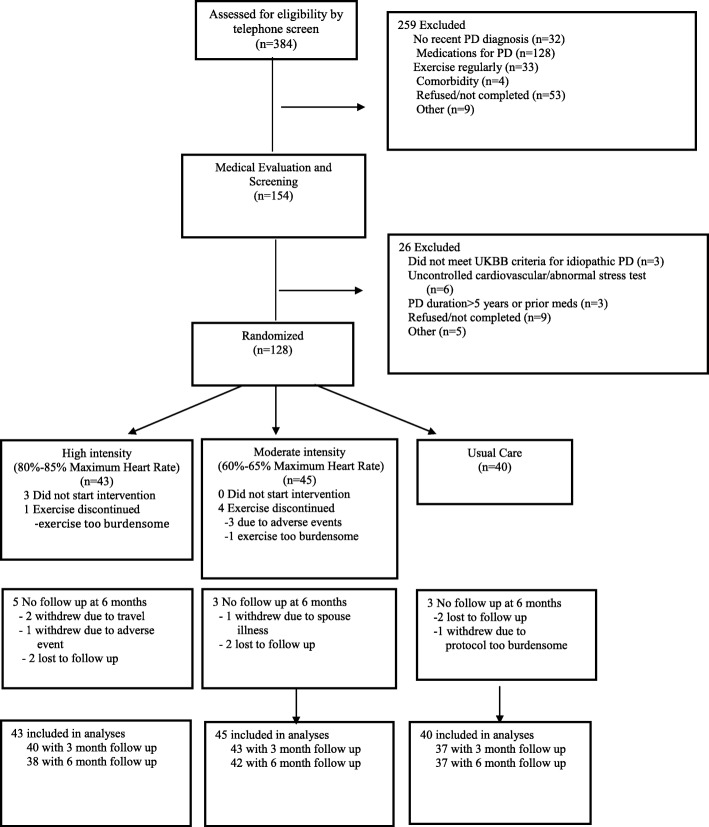
*Flow diagram* of patient participation in the study for the first six months

The purpose of this paper is to illustrate successful strategies employed by an academic movement disorder program to recruit patients with de novo PD for a one-year treadmill exercise study conducted at three sites. “De novo” is defined as the individual not yet taking prescribed PD-specific medications. The methods of the exercise study have been previously reported [4]. In brief, a Phase II, multi-center, randomized, controlled, futility trial investigating dose of exercise was planned with three groups; Group 1 performed treadmill exercise at high intensity four days weekly for 30 min; Group 2 performed treadmill exercise at moderate intensity four days weekly for 30 min; and Group 3 waited for six months and maintained prior levels of exercise, then were randomized to moderate versus high intensity treadmill exercise for the remaining six months (Fig. 1). All patients had PD within five years of diagnosis and were not taking PD medication at the start of the study. Primary feasibility measures were percentage of maximum heart rate and adherence. The primary futility measure was the six-month change in the motor score from the Unified Parkinson’s Disease Rating Scale. The results of the study demonstrated feasibility and safety of high intensity endurance exercise for people not yet on medications for PD and indicated that a Phase III investigation was warranted [5]. The highest enrolling site in this study recruited 76 patients with de novo PD into the clinical trial over a three-year recruitment period. The recruitment strategies used at this single site will be described below using four critical domains that are important in the recruitment process [1, 6].

## Methods

### Recruitment strategies

There are four critical domains that have been previously reported to be influential to recruitment: infrastructure; nature of the research; recruiter characteristics; and patient characteristics [1]. The strategies that will be described were performed at a tertiary movement disorder clinic that had nine movement disorder neurologists, two movement disorder fellows, two nurse study coordinators, two non-nurse study coordinators, two research assistants, a research administrator, videographer, database technician, and database manager. The movement disorder neurologists saw approximately 7000 patients per year, with 43% of them having PD.

#### Infrastructure

The need to access all potentially eligible participants is a key factor for successful recruitment. Infrastructure recruitment strategies that were used in the PD exercise RCT included fast-tracking de novo scheduling, weekly research meetings, use of a clinical repository, real-time clinic recruitment, and flyer postings.

1. Fast-track scheduling: All new patients to the clinic were scheduled through a centralized process within the Department of Neurology. After Institutional Review Board (IRB) approval of the exercise RCT, clinic schedulers were trained to ask prospective patients with PD if they had been treated with PD medication. If the patient had not, the patient was labeled “de novo” and one of the two study neurologists was contacted to see the patient. Patients with de novo PD were “fast-tracked”: seen within one week for a clinical evaluation, with many patients seen within 48 h. Patients confirmed to have de novo PD by the investigator, not requiring PD medication, and meeting inclusion criteria were recruited into the exercise RCT if they were interested in enrolling.
2. Research meetings: Meetings within the clinic occurred every Monday morning in order to review the current roster of recruiting studies, the numbers of enrolled individuals, and deadlines to reach recruitment milestones. All neurologists, fellows, study coordinators, and research assistants attended the meeting. PD studies were discussed by category of PD (such as de novo), inclusion and exclusion criteria were reviewed, number of individuals enrolled updated, and the number needed to fill the study were confirmed. Additional up-to-date reports from the study coordinator and/or research assistant and the study principal investigator (PI) were provided. Based on this process, the PD exercise RCT was discussed at the meeting at least twice monthly, with reminders to the faculty to refer any patients with de novo PD to the study team.
3. Clinical repository: Each new patient was consented for enrollment into an IRB-approved repository and videotaped [7]; the treating neurologist completed a database form with basic clinical information (Additional file 1). The data from the form were entered into a database, which could be quickly queried to ascertain specified patient populations for studies that were IRB-approved. Patients who enrolled in the repository were asked whether they agreed to be contacted for future studies. In addition to investigator data pulls, the database manager could also program the system to provide a research study alert sheet (“yellow form”) that was attached to each follow-up patient chart for those individuals in the repository who qualified for a particular study. This prompted the treating neurologist to recruit the patient for the identified study. Database pulls and study alert sheet were used to identify patients with de novo PD as they were seen in the clinic.
4. Real-time clinic recruitment: The physical space of the clinic consisted of staff and faculty offices that were contiguous with the clinic rooms so that the patient could be recruited at the time of their clinical visit. For this PD exercise RCT, the majority of the patients met with a study coordinator, research assistant, or PI at the time of their clinical appointment. A patient information form/consent form was given to the individual with follow-up via phone by the study coordinator within one week. In addition, flyer postings (IRB-approved) were used in the waiting room and in clinic exam rooms. This captured additional patients at the time of their visit.

#### Nature of the research

Several items were built into the PD exercise RCT that facilitated recruitment and retention. First, the design of the study with a wait-listed control exercise group improved the ability of the study team to recruit. Potential participants were more likely to enroll knowing that all individuals would be randomized to an exercise intervention at either the start of the study or at six months. Second, participants were trained initially at the academic center, then released to exercise at their local fitness center. This removed the need to travel to a busy downtown location for the exercise intervention. Third, participants were offered paid memberships to a local fitness center equipped with the treadmill needed for the exercise intervention. Last, heart rate monitors, which were included to track daily activity, were collected at home or shipped back to the study coordinator and eliminated additional monthly visits in the clinic. Recruitment difficulty is directly related to the extent of commitment required from the study itself, with randomized RCTs and, specifically, physical therapy studies being a more difficult recruitment in PD populations. The two site neurologists involved in this study were experienced in recruitment of patients with PD for PD exercise intervention studies. In order to facilitate recruitment for this study, the study was presented to the other faculty members in a presentation format at the weekly research meeting. This included the background/preclinical data, study hypothesis, design, inclusion and exclusion criteria, and goal recruitment. The study neurologists presented an overview of data supporting exercise in PD, safety of treadmill training, and justification of the chosen study arms. The study neurologists also provided key talking points regarding exercise for PD patient clinic interactions. There were some issues that negatively impacted recruitment that should be examined. The screening of the participants required a treadmill stress test. This procedure is not done in all PD exercise studies and caused a number of delays within the flow of the study. For those participants with a positive stress test, clearance was required from a primary care doctor or cardiologist and this increased the burden on the participant, which added a layer of complexity to the baseline visit. A second challenge in this study was that the main site for the study visits was in a busy, urban downtown location. Many of the potential participants lived in the suburbs or out of state (Table 1) and this location may have hindered patients from agreeing to participate despite the strategies described above. Last, criteria for this study excluded patients who had high levels of exercise at baseline. Although this was not an issue for this predominantly Midwestern population, this may be an issue for other geographic areas.

**Table 1:** Demographics of the participants

Age (years)	62.6 ± 9.7
Sex (% women)	34
Race (%)	
White	88
Black	4
Asian	0
Not reported	6
Ethnicity (% Hispanic)	8
Body mass index (kg/m^2^)	27.5 ± 4.5
Time since symptoms started (years)	2.3 ± 1.8
Time since diagnosis (years)	0.8 ± 0.9
Hoehn &Yahr Stage II (%)	100
Montreal Cognitive Assessment	27.7 ± 1.1
Unified Parkinson Disease Rating Scale Score	
Total	23.3 ± 8.2
Motor	16.5 ± 6.6
Activities of daily living	6.2 ± 3.6
Parkinson Disease Quality of Life Score	7.6 ± 5.7
Step count total (average daily)	5498 ± 2886
Geographic distance from study site (n)	
In Chicago	18
Within 15 miles of Chicago	24
> 15 miles from Chicago	34

#### Recruiter characteristics

It is widely reported that patients are more likely to agree to participate in research if they are asked by a medical doctor and that successful recruitment is frequently a team effort [1]. This PD exercise RCT had two movement disorder neurologists who shared responsibilities on the study, which allowed for fast-tracking clinic patients with de novo PD, coverage of in-person clinic recruitment, and greater flexibility in scheduling in-person assessments. In addition, the assigned nurse study coordinator had a 25-year history working with patients with PD and had a successful history of recruiting patients with de novo PD. She was frequently available after hours to talk to possible participants, would attend symposia and recruiting events, and was willing to contact participants using social media or texting. Midway through the PD exercise RCT, one of the study neurologists joined a NIH study designed to increase minority enrollment of patients with de novo PD into a second PD clinical trial. This study required extensive training in the recruitment of minority individuals; after completion, the study team was able to increase minority enrollment into the PD exercise RCT as well. Although it is argued whether it is possible to teach the “art of recruitment,” the experience of the current study team may have played a role.

#### Patient characteristics

The decision to take part in a study relies on three principal domains: (1) altruism; (2) personal health benefit; and (3) patient’s trust [5]. Personal health benefit may have been the largest contributor to recruitment in this exercise study, given the wealth of data that supports exercise as a therapeutic intervention in both healthy individuals and in PD. In addition, many of the patients received their clinical care in the recruiting clinic and this established relationship may have increased the patient’s trust and their likelihood to enroll. The design of the study may have increased the likelihood of recruitment as this exercise study enrolled patients with de novo PD. This patient population, as opposed to later stage patients, is more likely to be able to complete a long duration, labor-intensive exercise intervention. However, the patient characteristics were consistent across sites so this is not likely to have been a major contributor to the success at a single site. Patients with de novo PD need to be captured early in the disease course and may present to community neurologists initially. Two senior faculty members phoned general neurologists in the community who had previously referred patients to the program and described the PD exercise RCT and inclusion criteria. These calls yielded several de novo participants from two practices. Each community physician also received a letter from the study team when the individual enrolled, thanking them for the referral and detailing the specifics of the study. As part of the community outreach, faculty members hosted a yearly movement disorder symposium referring neurologists and patients, that typically drew 150–200 patients with PD, some of which are treated in the community. Research studies were highlighted as part of this program. Further outreach was done through several PD-related events, including Michael J. Fox Foundation events, during which recruitment occurs. In addition, a quarterly “PD101” symposium was started, specifically geared towards patients with early stage PD. A database pull identified patients who were within three years of disease onset and those patients were sent a personal invitation. Patients were also referred to PD101 by their treating neurologist. Data regarding the PD exercise RCT were included in the 3-h free seminar and the PI was available at the seminar to recruit.

## Results

The strategies described resulted in the recruitment of 76 patients with de novo PD. Demographics are located in Table 1 and successful recruitment strategies located in Table 2.

**Table 2:** Recruitment strategies and reason for success

Recruitment strategy	Reason for success
Fast-track scheduling	Shortened time for entry into the clinic and subsequently to recruitment
Research meeting recruitment reviews	Reminder to clinicians of the study entry criteria every Monday morning before the week’s clinics, increasing real-time recruitment
Clinical repository enrollment	Allowed creation of reminder sheets for recruitment into the exercise study at the time of the patient’s clinic visit
Contiguous clinic and research space	Study coordinators could meet with the potential subject immediately at the time of the clinic visit
Wait-listed control group	All patients knew that they would be receiving an exercise intervention
Local exercise and paid gym memberships	Patients could exercise locally and reduce the need to drive into a high traffic urban center
Heart rate monitors could be mailed back to study team	Alleviated the need for patients to travel back to the research clinic to return equipment
Multiple neurologists on the study	Allowed for in-person recruitment and more options for research visit scheduling at all times by a study neurologist
Experienced RN study coordinator	Facilitated recruitment by multiple techniques: support groups, social media, and after hours
Focused minority recruitment training	Raised investigator awareness and resulted in increased numbers of minorities recruited
De novo PD population	May be more motivated to participate in an intensive one-year exercise study due to a milder phenotype
Outreach to local neurologists	Increased the numbers of patients referred from the community for recruitment
Parkinson disease 101 course started	Focus on de novo Parkinson disease allowed for targeted recruitment at the quarterly seminars

## Conclusions

Suboptimal recruitment for RCTs can be a substantial barrier in the study and approval of new therapies for PD. This paper summarizes strategies that were used at an academic site to recruit 76 patients with de novo PD for an exercise RCT over a three-year period. This recruitment is high for exercise studies in PD. There have been only a few other studies that have successfully recruited this number of patients. Our group recruited 121 patients with PD who were on PD medications in a prior study for a 16 month study investigating flexibility training or aerobic training compared to home exercises, using many of the same recruitment methods described in this paper [8]. Eighty patients were recruited for a study investigating treadmill exercise at high and low intensity compared to stretching for a three-month study and is most similar to our study, albeit shorter in length [9]. Ninety-three patients were recruited for a treadmill study compared to controls for six months, but only 34 participants were available at the end of the study for analysis [10]. Finally, 105 patients were recruited for a recent exercise in PD study; however, the intervention was a minimally supervised exercise program [11]. There have been an additional 26 studies published since 2012 investigating exercise in PD, with an average recruitment of 40 participants per study, [12] making our studies in the high end of what has been reported in the past.

A focus on the infrastructure of the research program, experience of the study team, and ease of participation for the individuals were key players in the success of this PD exercise RCT. Several features of the study design also enhanced the ability of the study team to recruit. Although the strategies described here may not be feasible within all movement disorder programs, it is possible that some or all of them could be adopted or adapted accordingly.

## Additional file

Additional file 1: Database form for clinical information. (JPG 245 kb)
