# Supplementary figures and images for: Recruitment of patients with de novo Parkinson disease: successful strategies in a randomized exercise clinical trial
Source: Trials. 2018 Nov 14;19:630. doi: 10.1186/s13063-018-2958-z (PMC6237042; doi:10.1186/s13063-018-2958-z)

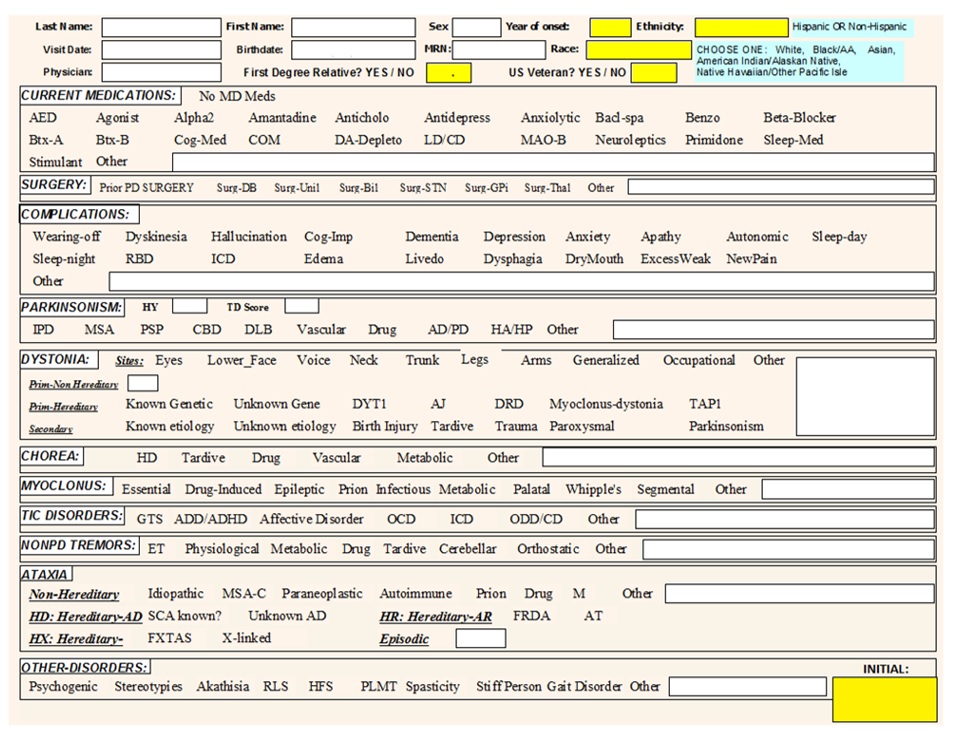

Supplement: Supplementary file 1 — Database form for clinical information. (JPG 245 kb) [file 13063_2018_2958_MOESM1_ESM.jpg]
